# Polycystic Ovary Syndrome Is Associated with Fatty Pancreas Disorder: A Case–Control Study in Non-Diabetic Women

**DOI:** 10.3390/jcm15176772

**Published:** 2026-08-31

**Authors:** Halil Severoglu, Fehmi Ateş, Huseyin Durukan

**Affiliations:** 1 Department of Internal Medicine, Faculty of Medicine, Mersin University, 33343 Mersin, Türkiye; 2 Department of Gastroenterology, Faculty of Medicine, Mersin University, 33343 Mersin, Türkiye; 3 Department of Obstetrics and Gynecology, Faculty of Medicine, Mersin University, 33343 Mersin, Türkiye

**Keywords:** polycystic ovary syndrome, fatty pancreas disorder, non-alcoholic fatty liver disease, metabolic syndrome, shear wave elastography, lipid accumulation product, hepatic steatosis index

## Abstract

**Background/Objectives:** Polycystic ovary syndrome (PCOS) is closely associated with insulin resistance, metabolic syndrome (MetS), and non-alcoholic fatty liver disease (NAFLD); however, whether fatty pancreas disorder is also more common in PCOS remains insufficiently clarified. This study aimed to compare the frequency of hepatic and fatty pancreas disorder and tissue stiffness between women with PCOS and a healthy control group and to determine the relationship of these findings with metabolic and biochemical parameters. **Methods:** In this retrospective case–control study, 40 women diagnosed with PCOS according to the 2003 Rotterdam criteria and 40 age- and body mass index (BMI)-matched healthy women were evaluated by abdominal ultrasonography and two-dimensional shear wave elastography (2D-SWE) of the liver and pancreas. Four validated non-invasive indices of hepatic steatosis (NAFLD liver fat score, lipid accumulation product [LAP], hepatic steatosis index [HSI], FIB-4, APRI) were calculated and correlated with clinical, anthropometric, and biochemical data. MetS was defined according to International Diabetes Federation (IDF) criteria. **Results:** Fatty pancreas disorder was detected in 47.5% of PCOS patients versus 17.5% of controls (*p* = 0.008); hepatic steatosis was found in 37.5% and 15%, respectively (*p* = 0.04); these differences were observed despite similar age and BMI between groups. LAP and HSI scores were higher in the PCOS group (*p* = 0.02 and *p* = 0.005, respectively), as were serum triglyceride and total testosterone levels (*p* = 0.001 and *p* = 0.005, respectively). Overall MetS frequency did not differ between the PCOS and control groups (*p* = 0.61) but was significantly higher among participants with fatty pancreas disorder (*p* = 0.009) and hepatic steatosis (*p* = 0.05). Hepatic and pancreatic 2D-SWE values were significantly higher in participants with steatosis than in those without (*p* = 0.02 and *p* = 0.04, respectively), and both correlated positively with BMI (*p* < 0.001 and *p* = 0.003, respectively). **Conclusions:** The frequency of fatty pancreas disorder and hepatic steatosis is increased in women with PCOS independent of obesity, and both are associated with a higher frequency of MetS; the association between fatty pancreas disorder and PCOS remained significant in a multivariable analysis adjusted for BMI. Fatty pancreas disorder showed a stronger association with MetS, suggesting that it may serve as an early, low-cost, ultrasound-detectable marker of metabolic risk in PCOS and warrants systematic evaluation alongside hepatic steatosis.

## 1. Introduction

Polycystic ovary syndrome (PCOS) is the most common endocrine disorder among women of reproductive age. Diagnosis is based on the Rotterdam criteria, which require at least two of three criteria to be present [1,2].

*PCOS was renamed “polyendocrine metabolic ovarian syndrome” (PMOS) following an international consensus process in May 2026 [3]; for continuity with the existing literature and reader familiarity, the established term “PCOS” is retained throughout this article.*

The 2003 Rotterdam consensus defines PCOS as the coexistence of chronic oligo- or anovulation, clinical and/or biochemical hyperandrogenism, and polycystic ovarian morphology on ultrasonography [4].

Both genetic and environmental factors contribute to the etiology of PCOS. Insulin resistance and its accompanying compensatory hyperinsulinemia impair ovarian folliculogenesis and increase ovarian androgen production, which in turn worsens insulin resistance and drives the clinical and biochemical manifestations of hyperandrogenism [5,6].

Androgen excess and ovarian dysfunction in PCOS are accompanied by disruption of the hypothalamic–pituitary–ovarian axis; increased luteinizing hormone (LH) pulsatility and an elevated LH/follicle-stimulating hormone (FSH) ratio provide the substrate for oligo- or anovulation [7].

PCOS is associated with a broad range of complications, including infertility, obesity, insulin resistance, type 2 diabetes mellitus, metabolic syndrome (MetS), dyslipidemia, hypertension, cardiovascular disease, and psychological morbidity; non-alcoholic fatty liver disease (NAFLD) and central obesity are also among these complications [7,8,9,10].

*As a recent terminological shift, an international consensus has proposed replacing the term ‘non-alcoholic fatty liver disease (NAFLD)’ with ‘metabolic dysfunction-associated steatotic liver disease (MASLD)’, which defines the disease according to underlying metabolic dysfunction rather than alcohol use [11]; throughout this article, the established term ‘NAFLD’ is retained for continuity with the existing literature (particularly the majority of the cited studies) and reader familiarity.*

NAFLD is the most common liver disease worldwide (prevalence of approximately 25%) and is defined as fat accumulation in more than 5% of hepatocytes in the absence of significant alcohol consumption [12,13]. Histologically similar to alcoholic liver disease, it can present a spectrum ranging from simple steatosis to lipotoxicity, steatohepatitis (NASH), and fibrosis [13,14]; progression to cirrhosis can be fatal [14]. NAFLD frequently coexists with obesity, type 2 diabetes, dyslipidemia, cardiovascular disease, MetS, and PCOS, conditions that share overlapping pathophysiological mechanisms [14,15,16].

Fatty pancreas disorder refers to fat infiltration of the pancreatic parenchyma. Its etiology is multifactorial, with MetS, alcohol use, viral infections, toxins, type 2 diabetes, male sex, advanced age, congenital syndromes, obesity, and certain medications all playing a role; obesity is considered the most important contributing factor [17,18]. Obesity and insulin resistance promote ectopic fat accumulation in the pancreas and other organs in parallel with NAFLD [19]. The mechanism underlying this relationship is thought to involve the release of free fatty acids and pro-inflammatory adipokines into the circulation once the limited storage capacity of dysfunctional adipose tissue is exceeded, which in turn triggers lipotoxicity, chronic low-grade inflammation, and insulin resistance; this process can lead to simultaneous ectopic fat accumulation in multiple organs, including the liver, pancreas, heart, and skeletal muscle. The recently described concept of a ‘metabolic steatotic axis’ offers a contemporary mechanistic framework linking adipose tissue dysfunction to this multi-organ steatosis, supporting the view that pancreatic steatosis should be regarded not as an isolated finding but as part of systemic metabolic dysfunction [20]. The recently convened Melbourne Consensus, bringing together 25 experts from six continents, formally defined this entity as “fatty pancreas disorder” (FPD) and emphasized that magnetic resonance imaging (MRI) is the preferred method for assessing intrapancreatic fat deposition [21]; in this article, the term “fatty pancreas disorder” is adopted in place of “pancreatic steatosis” to maintain terminological consistency with this consensus.

This study aimed to assess hepatic and fatty pancreas disorders and tissue stiffness in women with PCOS compared with a healthy control group using ultrasonography and two-dimensional shear wave elastography (2D-SWE) to examine non-invasive hepatic indices (NAFLD liver fat score, LAP, HSI, FIB-4, APRI) and to determine the relationship of these findings with metabolic parameters and biochemical measurements.

## 2. Materials and Methods

### 2.1. Study Design, Setting, and Ethical Approval

This retrospective, analytical case–control study was conducted using the records of women who presented to the Obstetrics and Gynecology, Endocrinology, and Gastroenterology outpatient clinics of Mersin University Faculty of Medicine Hospital between 1 June 2020 and 1 June 2021. The study protocol was approved by the Mersin University Faculty of Medicine Clinical Research Ethics Committee (approval no. 2021/626, dated 22 September 2021) and was conducted in accordance with the Declaration of Helsinki. Due to the retrospective design of the study, the requirement for individual written informed consent was waived by the ethics committee.

### 2.2. Participants and Eligibility Criteria

Women aged 18–44 years who were diagnosed with PCOS according to the 2003 Rotterdam criteria and who underwent abdominopelvic ultrasonography together with 2D-SWE elastography for assessment of hepatic and fatty pancreas disorder were included in the study. Healthy women in the same age range without a diagnosis of PCOS who underwent the same ultrasonographic and elastographic evaluation constituted the control group. A total of 40 women with PCOS and 40 controls (*n* = 80) were included in the study.

Exclusion criteria were as follows:

- Age outside the 18–44-year range;
- Polycystic ovarian morphology or clinical presentation not meeting the 2003 Rotterdam criteria;
- Pregnancy or lactation;
- Incomplete medical records;
- Diagnosis of diabetes mellitus;
- Previously known hepatic steatosis;
- Malignancy;
- Acute or chronic pancreatitis;
- Previous pancreatobiliary surgery;
- Use of metformin, oral contraceptives, anti-androgens, or lipid-lowering therapy;
- Alcohol consumption exceeding 20 g/day.

### 2.3. Sample Size Calculation

The study sample size was calculated based on the comparison of fatty pancreas disorder frequencies between women with and without PCOS. Sample size was determined using Cohen’s effect size approach for the difference between two independent proportions. Using Cohen’s effect size of h = 0.50 [22], a two-sided type I error rate of 5%, a power of 80%, and a 1:1 allocation ratio between groups, the required sample size was calculated as 40 per group for a total of 80 participants. Accordingly, a total of 80 participants—40 women with PCOS and 40 healthy controls—were included in the study.

### 2.4. Clinical, Anthropometric, and Biochemical Assessment

At presentation, the following data were recorded for all participants and controls: age, presence of hirsutism, Ferriman–Gallwey score, acne, oily skin, hair loss, infertility, menstrual pattern, waist circumference, hip circumference, height, weight, BMI, blood pressure, alcohol use, medication use, and diabetes status. Venous blood samples obtained after a 12 h fast on cycle days 2–3 were used to measure fasting glucose, insulin, 17-hydroxyprogesterone, dehydroepiandrosterone sulfate (DHEAS), progesterone, total testosterone, estradiol, prolactin, LH, FSH, total and direct bilirubin, platelet count, low-density lipoprotein (LDL) and high-density lipoprotein (HDL) cholesterol, triglycerides, aspartate aminotransferase (AST), alanine aminotransferase (ALT), gamma-glutamyl transferase (GGT), albumin, amylase, and lipase. Hepatic echogenicity (degree of steatosis and tissue stiffness) and pancreatic echogenicity (degree of steatosis and tissue stiffness) were also recorded.

### 2.5. Definitions and Calculated Indices

Insulin resistance was assessed using the homeostatic model assessment of insulin resistance (HOMA-IR) index, calculated as fasting insulin (mIU/L) × fasting glucose (mg/dL)/405; values above 2.6 were considered indicative of insulin resistance. BMI was calculated as weight (kg)/height^2^ (m^2^), and the waist-to-hip ratio was also calculated. Biochemical hyperandrogenism was defined as a serum total testosterone level above 0.6 ng/mL (2.08 nmol/L); clinical hyperandrogenism was assessed based on hirsutism (Ferriman–Gallwey score), androgenic alopecia, acne, and infertility.

MetS was defined according to International Diabetes Federation (IDF) criteria: central obesity (waist circumference ≥ 94 cm in European men or ≥80 cm in European women) plus at least two of the following: triglycerides ≥ 150 mg/dL; HDL cholesterol < 40 mg/dL in men or <50 mg/dL in women; blood pressure ≥ 130/85 mmHg; and fasting glucose > 100 mg/dL or a known diagnosis of type 2 diabetes [23,24].

Hepatic steatosis was additionally assessed using four validated non-invasive indices: the NAFLD liver fat score, calculated as −2.89 + 1.18 × (MetS present = 1, absent = 0) + 0.45 × (type 2 diabetes present = 2, absent = 0) + 0.15 × fasting insulin (mU/L) + 0.04 × AST (U/L) − 0.94 × (AST/ALT) [25]; the lipid accumulation product (LAP), calculated in women as (waist circumference [cm] − 58) × triglycerides (mmol/L) [26]; and the hepatic steatosis index (HSI), calculated as 8 × (ALT/AST) + BMI (kg/m^2^) + 2 (if female) + 2 (if type 2 diabetes present, otherwise 0) [27]. Hepatic fibrosis was estimated using the FIB-4 index ((age [years] × AST [U/L])/(platelet count [×10^9^/L] × √ALT [U/L])) [28] and the AST-to-platelet ratio index (APRI) (100 × (AST/upper limit of normal AST [U/L])/platelet count (×10^9^/L)) [29].

### 2.6. Ultrasonographic and Elastographic Assessment

All ultrasonographic examinations were performed by the same experienced gastroenterologist, who was blinded to whether participants belonged to the control or PCOS group. Hepatic steatosis was graded by comparing hepatic parenchymal echogenicity with renal and splenic echogenicity: grade 1 (mild), diffusely increased hepatic echogenicity with normal visualization of the diaphragm and vessel walls; grade 2 (moderate), diffusely increased echogenicity with impaired visualization of vessel walls and the diaphragm; and grade 3 (severe), markedly increased echogenicity with poor visualization of the diaphragm and accompanying posterior attenuation. Fatty pancreas disorder was graded according to pancreatic echogenicity as follows: grade 1 (mild), pancreatic echogenicity slightly increased relative to the liver, with clear visualization of pancreatic borders and the splenic vein; grade 2 (moderate), pancreatic echogenicity markedly higher than hepatic echogenicity but lower than retroperitoneal fat, with blurred visualization of pancreatic borders; and grade 3 (severe), pancreatic echogenicity equal to or greater than retroperitoneal fat, with indistinct pancreatic borders and a non-visualized splenic vein.

Hepatic and pancreatic tissue stiffness were measured in kilopascals (kPa) using two-dimensional shear wave elastography (2D-SWE), in which shear wave propagation velocity is directly proportional to tissue stiffness. Ten consecutive measurements were obtained for each organ and averaged. A high-resolution ultrasonography device (Toshiba Applio 500, Toshiba Medical Systems Corporation, Tokyo, Japan) was used for all elastographic measurements.

### 2.7. Statistical Analysis

Continuous variables were presented as mean ± standard deviation and minimum–maximum values, and categorical variables as frequency and percentage. Depending on the distributional characteristics of continuous variables, Student’s *t*-test was used for between-group comparisons of normally distributed variables and the Mann–Whitney U test for non-normally distributed variables (including lipase). Associations between categorical variables were assessed using the chi-square test. Associations between continuous variables were assessed using Pearson’s correlation coefficient. A two-sided *p* < 0.05 was considered statistically significant. Statistical analyses were performed using MedCalc statistical software, version 23.0.2 (MedCalc Software Ltd., Ostend, Belgium). 

During the preparation of this manuscript, the author(s) used Claude (Anthropic) for the purposes of language editing and formatting. The author(s) have reviewed and edited the output and take full responsibility for the content of this publication.

## 3. Results

A total of 80 women aged 18–44 years were included in the study; 40 had PCOS and 40 were healthy controls. Mean BMI was 23.33 ± 3.02 kg/m^2^ in the PCOS group and 22.52 ± 3.38 kg/m^2^ in the control group; there was no significant difference between groups in age or BMI (*p* > 0.05).

### 3.1. Baseline Sociodemographic and Clinical Characteristics

The sociodemographic and clinical characteristics of the two groups are summarized in Table 1.

Systolic blood pressure was significantly higher in women with PCOS than in controls (114.65 ± 13.91 vs. 108.2 ± 11.5 mmHg, *p* < 0.05). No significant differences were observed between groups in BMI, diastolic blood pressure, waist circumference, hip circumference, or waist-to-hip ratio (all *p* > 0.05).

Menstrual irregularity was significantly more frequent in the PCOS group than in controls (72.5% vs. 15%, *p* < 0.05); hirsutism (50% vs. 7.5%, *p* < 0.05), acne (55% vs. 17.5%, *p* < 0.05), and a Ferriman–Gallwey score >8 (57.5% vs. 7.5%, *p* < 0.05) were also more frequent. There were no significant differences between groups in oily skin, infertility, hair loss, BMI category (≥25 vs. <25 kg/m^2^), or presence of insulin resistance (*p* > 0.05).

### 3.2. Hormonal and Biochemical Parameters

Laboratory and biochemical parameters are shown in Table 2.

Total testosterone (0.52 ± 0.19 vs. 0.39 ± 0.19 ng/mL, *p* < 0.05), triglycerides (90.6 ± 39.37 vs. 56.17 ± 13.68 mg/dL, *p* < 0.05), LAP score (15.37 ± 14.75 vs. 9.52 ± 5.62, *p* < 0.05), and HSI score (47.12 ± 0.74 vs. 42.9 ± 0.68, *p* < 0.05) were significantly higher in PCOS patients than in controls. No significant differences were found in fasting glucose, insulin, HOMA-IR, DHEAS, LH, FSH, LDL, HDL, platelet count, GGT, albumin, AST, total cholesterol, amylase, lipase, NAFLD score, FIB-4, or APRI (all *p* > 0.05); hepatic and pancreatic elastography values were also similar between groups (*p* > 0.05).

### 3.3. Frequency of Hepatic Steatosis, Fatty Pancreas Disorder, and Metabolic Syndrome

The frequency of hepatic steatosis, fatty pancreas disorder, and MetS in the two groups is shown in Table 3.

Hepatic steatosis and fatty pancreas disorder were markedly more frequent in women with PCOS than in controls; crude odds ratios were 3.40 and 4.27, respectively (Table 3), indicating that PCOS increases the risk of steatosis in both organs by approximately three- to four-fold. In contrast, steatosis severity (mild/moderate distribution) and overall MetS frequency did not differ significantly between groups (both *p* > 0.05).

### 3.4. Factors Associated with Hepatic Steatosis

Factors associated with the presence of hepatic steatosis, independent of PCOS status, are presented in Table 4.

Compared with participants without hepatic steatosis, those with hepatic steatosis were older (32 ± 8.5 vs. 27.5 ± 6.8 years, *p* < 0.05) and had higher BMI (26.16 ± 2.17 vs. 21.77 ± 2.71 kg/m^2^, *p* < 0.001), waist circumference (85 ± 9.64 vs. 70.98 ± 7.43 cm, *p* < 0.001), hip circumference (105.33 ± 7.46 vs. 95.35 ± 8.27 cm, *p* < 0.001), waist-to-hip ratio (0.8 ± 0.05 vs. 0.73 ± 0.06, *p* < 0.001), and serum triglyceride levels (85.61 ± 48.05 vs. 69.03 ± 26.59 mg/dL, *p* = 0.05). LAP (22.73 ± 15.95 vs. 8.79 ± 6.31, *p* = 0.001) and HSI (38.39 ± 3.14 vs. 34.6 ± 4.79, *p* = 0.001) scores were also higher in the presence of hepatic steatosis. Hepatic 2D-SWE values were higher in participants with hepatic steatosis than in those without (8.49 ± 2.21 vs. 7.42 ± 1.66 kPa, *p* = 0.02); pancreatic 2D-SWE values were similarly higher (8.95 ± 1.76 vs. 7.92 ± 1.27 kPa, *p* = 0.005). MetS (14.3% vs. 1.7%, *p* = 0.05) and fatty pancreas disorder (85.7% vs. 13.6%, *p* < 0.001) were also significantly more frequent in participants with hepatic steatosis. No significant association was found between hepatic steatosis and insulin resistance (*p* > 0.05).

### 3.5. Factors Associated with Fatty Pancreas Disorder

Factors associated with the presence of fatty pancreas disorder, independent of PCOS status, are presented in Table 5.

Participants with fatty pancreas disorder were older than those without (31.1 ± 8.1 vs. 27.6 ± 7.1 years, *p* = 0.04) and had higher BMI (25.71 ± 2.26 vs. 21.58 ± 2.71 kg/m^2^, *p* < 0.001), systolic blood pressure (117.08 ± 15.13 vs. 108.71 ± 19.12 mmHg, *p* = 0.02), waist circumference (81.46 ± 10.63 vs. 71.38 ± 8.13 cm, *p* < 0.001), hip circumference (103.04 ± 7.62 vs. 95.53 ± 8.88 cm, *p* < 0.001), waist-to-hip ratio (0.78 ± 0.07 vs. 0.74 ± 0.05, *p* = 0.001), and serum triglyceride levels (91.15 ± 46.55 vs. 64.83 ± 21.77 mg/dL, *p* = 0.01). In the presence of fatty pancreas disorder, pancreatic 2D-SWE values (8.31 ± 2.17 vs. 7.41 ± 1.64 kPa, *p* = 0.04) and hepatic 2D-SWE values (8.95 ± 1.77 vs. 7.83 ± 1.17 kPa, *p* = 0.006) were also higher; MetS frequency (15.4% vs. 0%, *p* = 0.009) and concomitant hepatic steatosis (69.2% vs. 5.6%, *p* < 0.001) were likewise more frequent. No significant association was found between insulin resistance and fatty pancreas disorder (*p* > 0.05).

### 3.6. Correlation of Elastography with Age and BMI

The correlation of hepatic and pancreatic 2D-SWE values with age and BMI is shown in Table 6.

Both hepatic and pancreatic elastography values correlated positively with BMI (r = 0.40, *p* < 0.001 and r = 0.35, *p* = 0.003, respectively), but were not associated with age (r = −0.12, *p* = 0.29 and r = 0.07, *p* = 0.53, respectively).

### 3.7. Multivariable Analysis of Factors Associated with Pancreatic Steatosis

Age, BMI, systolic blood pressure, triglycerides, and PCOS status—the variables found to be associated with fatty pancreas disorder on univariable analysis in Table 5—were entered into a multivariable logistic regression model with the presence/absence of fatty pancreas disorder as the dependent variable. Model results are presented in Table 7.

In the multivariable model, BMI (*p* < 0.001; Exp(B) = 1.765; 95% CI 1.323–2.354) and PCOS status (*p* = 0.048; Exp(B) = 5.474; 95% CI 1.018–29.426) were independently associated with fatty pancreas disorder; age, systolic blood pressure, and triglyceride level did not reach significance in this model (all *p* > 0.05). This finding supports that the association between PCOS and fatty pancreas disorder persists independently of general adiposity as expressed by BMI. Notably, the point estimate for PCOS status (Exp(B) = 5.474) was higher than that for BMI (Exp(B) = 1.765), suggesting that the association between PCOS and fatty pancreas disorder may be more pronounced than that of adiposity as expressed by BMI. However, because these two variables are measured on different scales (BMI being continuous and PCOS binary), direct comparison of their Exp(B) values should be made with caution; furthermore, the wide confidence interval for PCOS (1.018–29.426) reflects estimation uncertainty owing to the relatively small sample size, and this finding requires confirmation in larger cohorts.

## 4. Discussion

The new nomenclature was developed through a global consensus process involving 56 patient organizations and societies worldwide, including ASRM. The process included multiple workshops with women with lived experience and healthcare professionals, informed by a survey with 22,000 responses [3].

PCOS is a common endocrine disorder characterized by hormonal dysregulation and ovarian dysfunction, clinically manifesting as menstrual irregularity and hyperandrogenism. It is well established that PCOS contributes to abdominal adiposity, insulin resistance, obesity, female infertility, and the development of cardiovascular disease [6,30]. MetS itself is a major cardiovascular risk factor; its components—insulin resistance and accompanying hyperglycemia, obesity, hypertension, low HDL cholesterol, and high triglycerides—are closely intertwined with PCOS, and it is now widely accepted that PCOS predisposes to obesity, dyslipidemia, hypertension, and NAFLD [31]. When circulating free lipids exceed the storage capacity of adipose tissue, they begin to accumulate ectopically in other organs, particularly the pancreas, liver, heart, and skeletal muscle. Previous studies have shown that pancreatic fat accumulation develops before overt pancreatic dysfunction [32]. Singh et al., in a meta-analysis of 12,675 participants using magnetic resonance imaging, reported a prevalence of fatty pancreas disorder of approximately 33% in the general adult population [33]. The neurohormonal disturbances, chronic low-grade inflammation, insulin resistance, and compensatory hyperinsulinemia that characterize PCOS increase adipogenesis and reduce lipolysis, thereby promoting visceral adiposity [34]. Although these mechanisms would be expected to increase the frequency of fatty pancreas disorder in PCOS, no prior study has directly addressed this question. In our study, using ultrasonography and 2D-SWE, fatty pancreas disorder was found to be approximately three times more frequent in women with PCOS than in healthy controls (47.5% [19/40] vs. 17.5% [7/40], *p* = 0.008). Since BMI and waist-to-hip ratio were similar between the two groups (Table 1; BMI: 23.33 ± 3.02 vs. 22.52 ± 3.38 kg/m^2^, *p* = 0.26) and age distribution did not differ either, this difference may reflect the metabolic effects of PCOS itself. On the other hand, when participants were classified according to the presence of fatty pancreas disorder (Table 5), those with the disorder had significantly higher BMI, waist circumference, waist-to-hip ratio, systolic blood pressure, and triglyceride levels than those without, indicating that fatty pancreas disorder is closely intertwined with overall metabolic dysfunction. In the multivariable logistic regression analysis performed to assess the confounding effect of these associations (Table 7), PCOS status remained independently associated with fatty pancreas disorder after adjustment for BMI; however, this estimate comes with a wide confidence interval (95% CI 1.018–29.426), and adjustment could not be made for other potential confounders such as waist-to-hip ratio, insulin resistance, and androgen levels. The phrase ‘independent of obesity’ should therefore be interpreted cautiously and only within the context of this BMI-adjusted analysis. Hyperandrogenemia and hyperlipidemia frequently coexist in PCOS [35]; in our cohort, PCOS patients had higher plasma triglyceride and androgen levels than controls, which may partly explain the obesity-independent increase in fatty pancreas disorder we observed. This finding is also clinically important, as fatty pancreas disorder predisposes to type 2 diabetes and is a recognized risk factor for pancreatic cancer [36,37,38].

NAFLD has a rising prevalence worldwide and encompasses a spectrum ranging from simple steatosis to NASH, fibrosis, and cirrhosis; its diagnosis can be challenging, as it is often asymptomatic and does not always present with abnormal laboratory findings [15]. NAFLD is known to be more frequent in women with PCOS independent of age and BMI [16,39,40,41]. In our study, hepatic steatosis was found to be 2.5 times more frequent in PCOS patients with similar age, waist-to-hip ratio, and BMI compared with controls (37.5% [15/40] vs. 15% [6/40], *p* = 0.04; crude OR = 3.40, 95% CI 1.16–10.00), suggesting that this difference may be attributable to PCOS itself rather than to obesity. Consistent with our findings, Wu et al., in a meta-analysis, reported that NAFLD was approximately 2.3 times more frequent in women with PCOS than in controls, independent of obesity, and proposed that this association may be linked to hyperandrogenism [42]. The significantly higher serum total testosterone levels found in the PCOS group in our study (0.52 ± 0.19 vs. 0.39 ± 0.19 ng/mL, *p* = 0.005) support this hypothesis.

Liver biopsy remains the reference standard for the diagnosis of NAFLD; however, its invasive nature, cost, and susceptibility to sampling error have driven the development of non-invasive hepatic indices and imaging techniques [16]. In our study, the non-invasive hepatic steatosis indices of NAFLD score, LAP, and HSI were used together with the fibrosis indices of FIB-4 and APRI. FIB-4 and APRI, which primarily reflect advanced fibrosis and end-stage liver disease, did not differ between groups (*p* > 0.05); this is likely explained by the similarity between PCOS and control participants with respect to the variables determining these two scores, as well as the young age and short disease duration of the PCOS patients in our cohort.

In contrast, LAP (15.37 ± 14.75 vs. 9.52 ± 5.62, *p* = 0.02) and HSI (47.12 ± 0.74 vs. 42.9 ± 0.68, *p* = 0.005) scores were significantly higher in PCOS patients than in controls. The elevated LAP score in PCOS was consistent with the higher triglyceride levels in this group. HSI is a composite non-invasive index reflecting the risk of hepatic steatosis; hyperandrogenism has been shown in the literature to be associated with hepatic steatosis in women with PCOS independent of insulin resistance [43]. In our cohort, total testosterone levels were also significantly higher in the PCOS group than in controls, suggesting that the elevation in HSI score may be associated with hyperandrogenism independent of obesity. Chakraborty et al. similarly reported significantly higher NAFLD and LAP scores in women with PCOS compared with controls, but found no significant difference in HSI [44]; in contrast, in our cohort HSI was significantly elevated while the NAFLD score did not differ.

Growing evidence suggests that fatty pancreas disorder may serve as an early marker of the ectopic fat accumulation underlying both NAFLD and MetS [17]. In a meta-analysis, Bi et al. showed that non-alcoholic fatty pancreas disease was independently and significantly associated with both NAFLD (RR = 2.49) and MetS (RR = 2.25) (both *p* < 0.0001) [45]; Bhalla et al., in a retrospective cohort of 265 participants, identified age (*p* < 0.01), body mass index (*p* < 0.01), and hyperlipidemia (*p* < 0.05) as predictors of pancreatic fatty infiltration; in the subgroup of women, elevated body mass index was independently associated with pancreatic fat (*p* = 0.023), and the relationship between pancreatic fat and metabolic syndrome components was found to be sex-dependent [46]. In our cohort, overall MetS frequency did not differ significantly between the PCOS and control groups; however, when participants were classified according to the presence of fatty pancreas disorder or hepatic steatosis, MetS was significantly more frequent in both steatosis groups than in those without steatosis (*p* = 0.009 and *p* = 0.05, respectively). Participants with fatty pancreas disorder or hepatic steatosis were also older (both *p* = 0.04) and had higher BMI (both *p* < 0.001) than those without. This pattern is consistent with a sequence in which PCOS first promotes pancreatic and hepatic fat accumulation, followed by the development of MetS. The stronger association we observed between fatty pancreas disorder and MetS, compared with hepatic steatosis, suggests that it may be reasonable to include fatty pancreas disorder as a component of MetS assessment. The higher systolic blood pressure observed in participants with fatty pancreas disorder compared with those without (*p* = 0.02) is a further finding supporting the close relationship between fatty pancreas disorder and MetS.

Hyperandrogenism, characterized by systemically elevated androgen levels, is the hallmark feature of PCOS; its clinical manifestations include hirsutism, acne, androgenic alopecia, infertility, and menstrual irregularity. In our cohort, PCOS patients had significantly higher plasma triglyceride and total testosterone levels than the age- and BMI-matched control group (*p* = 0.005 and *p* = 0.001, respectively). Similarly, Hestiantoro et al., in a prospective study, found that PCOS patients with hyperandrogenism had significantly higher plasma triglyceride and testosterone levels than PCOS controls without hyperandrogenism of similar age and body weight (*p* = 0.01 and *p* = 0.04, respectively) [35].

Hirsutism, the most common clinical manifestation of hyperandrogenism, is assessed using the Ferriman–Gallwey score, a standardized hair-growth scoring system [47,48]. In our study, hirsutism, acne, oily skin, hair loss, and menstrual irregularity were significantly more frequent in PCOS patients than in controls, a pattern attributable to the higher androgen levels characteristic of PCOS.

Systolic blood pressure was also significantly higher in our PCOS patients than in controls (*p* = 0.03). Similarly, Mellembakken et al. reported higher systolic and diastolic blood pressure and total testosterone in women with PCOS compared with a control group of similar age and BMI (*p* < 0.001, *p* < 0.001, and *p* < 0.001, respectively) [49]. Elevated blood pressure is a component of MetS and a major risk factor for cardiovascular disease.

Participants with ultrasonographically detected hepatic steatosis had significantly higher BMI, waist-to-hip ratio, and triglyceride levels than those without (*p* < 0.001, *p* < 0.001, and *p* = 0.05, respectively), consistent with the frequent co-occurrence of MetS components with NAFLD. Similarly, participants with fatty pancreas disorder had significantly higher BMI, waist-to-hip ratio, systolic blood pressure, and triglyceride levels than those without (*p* < 0.001, *p* = 0.001, *p* = 0.02, and *p* = 0.01, respectively); this association appears stronger than that observed for hepatic steatosis. Participants with fatty pancreas disorder or hepatic steatosis were also older than those without (both *p* = 0.04), consistent with the view that the risk of MetS, NAFLD, and fatty pancreas disorder increases with age.

Ultrasound-based imaging offers wide bedside availability, relatively low cost, and a non-invasive profile. Shear wave elastography provides information on tissue stiffness: fibrosis and inflammation increase stiffness and reduce elasticity, although elastographic thresholds may vary according to comorbidities and the ultrasound system used [50,51,52]. Elastography has been widely used to assess tissue stiffness in NAFLD; Ochi et al. compared ultrasound elastography with liver biopsy in NAFLD patients and proposed elastography as a diagnostic tool for assessing hepatic fibrosis [52], and a study cited in the review by Chimoriya et al. reported 2D-SWE cut-off values of 8.9 kPa and 10.2 kPa for significant fibrosis (≥F2) and cirrhosis, respectively, in NAFLD [53]. Xiao et al. concluded that both magnetic resonance elastography and ultrasound-based SWE are reliable methods for assessing hepatic fibrosis [54]. In our study, hepatic 2D-SWE values were significantly higher in participants with hepatic steatosis than in those without (8.49 ± 2.21 vs. 7.42 ± 1.66 kPa, *p* = 0.02). Pancreatic elastography similarly allows assessment of pancreatic tissue stiffness: Sezgin et al. demonstrated increased pancreatic stiffness by ultrasound elastography in patients with acute pancreatitis [55], and a separate study found that pancreatic 2D-SWE values were significantly higher in the presence of fatty pancreas disorder and correlated positively with MetS and its components (all *p* < 0.001) [37]. Consistent with these findings, pancreatic 2D-SWE values in our participants were significantly higher in those with fatty pancreas disorder than in those without (8.31 ± 2.17 vs. 7.41 ± 1.64 kPa, *p* = 0.04). In our cohort, both fatty pancreas disorder and hepatic steatosis correlated positively with tissue stiffness and MetS; this association may reflect chronic low-grade inflammation predisposing to fibrosis in steatotic organs.

Obesity promotes pro-inflammatory changes in metabolically active cells such as adipocytes, hepatocytes, and myocytes and activates immune cells that secrete inflammatory cytokines such as TNF-α, IL-6, and adiponectin; sustained elevation of these cytokines can lead to chronic inflammation and ultimately fibrosis [34]. Consistent with this mechanism, we found a positive correlation between BMI and both hepatic and pancreatic tissue elastography values (*p* < 0.001 and *p* = 0.03, respectively).

In interpreting the observed associations, it is important to distinguish statistical significance from clinical significance. For example, although the differences in systolic blood pressure (117.08 ± 15.13 vs. 108.71 ± 19.12 mmHg) and triglyceride levels (91.15 ± 46.55 vs. 64.83 ± 21.77 mg/dL) between the PCOS and control groups were statistically significant, it remains unclear to what extent these absolute differences would alter clinical decision-making at the level of an individual patient, and these differences largely remain within normal-to-borderline ranges in both groups. Similarly, the significant but modest differences in hepatic and pancreatic elastography values (8.49 ± 2.21 vs. 7.42 ± 1.66 kPa and 8.95 ± 1.76 vs. 7.92 ± 1.27 kPa, respectively) should be carefully considered in relation to where established clinical tissue-stiffness thresholds lie. Our findings should therefore be interpreted not as decision-changing thresholds for direct use in clinical practice, but as signals warranting further investigation for metabolic monitoring in PCOS.

**Future Research Perspective:** Our findings are hypothesis-generating and require confirmation. In particular, prospective, multicenter cohort studies with large sample sizes, using standardized imaging protocols (preferably MRI-based), are needed to assess whether fatty pancreas disorder predicts incident metabolic syndrome, type 2 diabetes mellitus, MASLD progression, or cardiovascular outcomes in women with PCOS, independent of BMI. Such studies will clarify whether fatty pancreas disorder is a clinically meaningful early marker in PCOS and will lay the groundwork for the development of screening strategies.

### Strengths and Limitations of the Study

Strengths of our study include that it is, to our knowledge, the first study conducted in our country and one of few worldwide to assess hepatic and fatty pancreas disorder together in the same cohort of women with PCOS, alongside an age- and BMI-matched healthy control group. All ultrasonographic and elastographic assessments were performed by a single experienced gastroenterologist, blinded to group assignment, using a standardized protocol, thereby minimizing inter-observer variability.

The limitations of our study restrict the generalizability of the findings. The single-center design, the relatively small sample size (*n* = 80), the exclusion of patients with diabetes (meaning our findings cannot be generalized to women with PCOS and type 2 diabetes), and the assessment of pancreatic/hepatic steatosis by ultrasonography rather than by the gold-standard MRI-PDFF or histological confirmation are the principal factors limiting the transferability of our findings to different populations and clinical settings. Although the multivariable logistic regression model presented for fatty pancreas disorder (Table 7) demonstrates the independent contribution of BMI and PCOS status, it has wide confidence intervals owing to the relatively small sample size and did not allow the inclusion of additional potential confounders (e.g., waist-to-hip ratio, androgen levels, insulin resistance) in the model. Furthermore, numerous univariable comparisons were performed in our study without formal statistical correction for multiple testing (e.g., Bonferroni); accordingly, individual *p*-values, particularly those of borderline significance, should be interpreted cautiously in light of the risk of type I error.

Regarding medication use, participants using metformin, oral contraceptives, anti-androgens, or lipid-lowering therapy were excluded from both the PCOS and control groups (see Eligibility Criteria), and both groups were composed of medication-naïve participants; medication use therefore does not constitute a confounding factor in this cohort. On the other hand, the retrospective design of our study did not allow systematic documentation of PCOS phenotype subgroups, standardized grading of hyperandrogenism severity, lifestyle characteristics, or disease duration; as these variables may influence ectopic fat accumulation, our findings should be interpreted with these clinical details unaccounted for.

Hepatic and pancreatic biopsy, the reference standard for steatosis and fibrosis, was not performed, and elastographic threshold values were not independently validated against histology in this cohort. Abdominal ultrasonography, used to assess intrapancreatic fat deposition, also carries an important limitation of its own: the method is operator-dependent, has limited sensitivity for distinguishing mild degrees of steatosis, and image quality can frequently be degraded owing to the retroperitoneal location of the pancreas and bowel gas; furthermore, ultrasonographic grading is semi-quantitative and cannot numerically measure the proportion of intrapancreatic fat. Indeed, the Melbourne Consensus recommends magnetic resonance imaging (MRI), which allows voxel-based fat quantification, as the preferred method for assessing intrapancreatic fat deposition and does not regard conventional endoscopic ultrasonography as a validated method for this purpose [21]; the ultrasonography-based grading used in our study should therefore be considered to potentially have lower sensitivity than MRI-based measurements, and mild cases of fatty pancreas disorder may have been missed. Finally, because participants with a diagnosis of type 2 diabetes mellitus were excluded per the exclusion criteria, the contribution of this component of the NAFLD liver fat score could not be assessed.

**External Validity:** The generalizability of these findings is limited by the characteristics of the study population. Participants were recruited from a single tertiary referral center in Türkiye and consisted exclusively of premenopausal, non-diabetic, medication-naïve women diagnosed with PCOS according to the 2003 Rotterdam criteria; women meeting alternative diagnostic frameworks (e.g., NIH or AE-PCOS criteria), postmenopausal women, women with diabetes, and women receiving hormonal or metabolic therapy were not represented. The relatively homogeneous ethnic and geographic background of the cohort, combined with the single-center design and modest sample size, restricts the extent to which the observed frequencies of fatty pancreas disorder and hepatic steatosis, and their associations with PCOS, can be extrapolated to other populations, healthcare settings, or PCOS phenotypes. Confirmation in multi-center, prospective studies enrolling larger and more diverse cohorts is required before these findings can be considered broadly generalizable.

## 5. Conclusions

In our retrospective case–control study, the limited global data—and complete absence of data in our country—on fatty pancreas disorder and pancreatic tissue stiffness in women with PCOS, and their relationship with biochemical and clinical parameters, render the present findings clinically important. Our findings can be summarized as follows:

1. The frequency of fatty pancreas disorder is increased in women with PCOS, and this association remained significant after statistically controlling for the effect of BMI (Table 7); the point estimate for PCOS (Exp(B) = 5.474) was higher than that for BMI (Exp(B) = 1.765). This finding supports an increased risk of fatty pancreas disorder in women with PCOS, at least independent of BMI; however, given the wide confidence interval of the estimate and other potential confounders that could not be adjusted for (waist-to-hip ratio, insulin resistance, androgen levels), confirmation of this association in larger prospective cohorts is recommended, together with investigation of the benefit of early screening approaches in women with PCOS.
2. The frequency of hepatic steatosis is likewise increased in women with PCOS, independent of age, obesity, waist-to-hip ratio, insulin resistance, and MetS (crude OR = 3.40, 95% CI 1.16–10.00). Early screening is warranted in this population to prevent NAFLD-related morbidity and mortality.
3. Fatty pancreas disorder was more frequent than hepatic steatosis in women with PCOS. Fatty pancreas disorder should be recognized and addressed in these patients before pancreatic or systemic complications develop. These findings are pending confirmation in larger, multi-center cohorts.
4. Although overall MetS frequency did not differ between women with PCOS and the healthy control group, MetS frequency was significantly increased among participants with fatty pancreas disorder or hepatic steatosis, and this increase was more pronounced for fatty pancreas disorder. Fatty pancreas disorder may therefore serve as an early marker of evolving MetS.
5. Women with PCOS have higher triglyceride levels and systolic blood pressure than controls; these findings may be related to the hyperandrogenism characteristic of PCOS. As both parameters are components of MetS and contribute to cardiovascular and other systemic morbidity, triglyceride levels and blood pressure should be routinely measured in women with PCOS.
6. Ultrasound-based 2D-SWE demonstrated increased hepatic and pancreatic tissue stiffness in women with steatosis in these organs. As a non-invasive and low-cost method, 2D-SWE may be used to assess hepatic and pancreatic inflammation and fibrosis in this population.
7. The non-invasive hepatic steatosis indices LAP and HSI were elevated both in women with PCOS and in participants with hepatic steatosis. Given their ease of use, low cost, and non-invasive nature, wider use of these indices is recommended.

These findings are preliminary and it is recommended that they be supported by larger, multicenter, prospective studies.

## Figures and Tables

**Table 1:** Sociodemographic and clinical characteristics of the PCOS and control groups (*n* = 80).

Characteristic	Total (*n* = 80)	PCOS (*n* = 40)	Control (*n* = 40)	*p*-Value
Age (years), mean ± SD	28.7 ± 7.5	28.4 ± 7.5	29.1 ± 7.6	0.69
Menstrual irregularity, *n* (%)	35 (43.8)	29 (72.5)	6 (15)	<0.001
Hirsutism, *n* (%)	23 (28.7)	20 (50)	3 (7.5)	<0.001
Acne, *n* (%)	29 (36.3)	22 (55)	7 (17.5)	<0.001
Oily skin, *n* (%)	29 (36.3)	16 (40)	13 (32.5)	0.48
Infertility, *n* (%)	1 (1.3)	1 (2.5)	0 (0)	0.51
Hair loss, *n* (%)	53 (66.3)	24 (60)	29 (72.5)	0.24
Ferriman–Gallwey score >8, *n* (%)	26 (32.5)	23 (57.5)	3 (7.5)	<0.001
Insulin resistance, *n* (%)	9 (11.3)	4 (10)	5 (12.5)	0.72
BMI ≥ 25 kg/m^2^, *n* (%)	23 (28.7)	13 (32.5)	10 (25)	0.5
BMI (kg/m^2^), mean ± SD	22.93 ± 3.21	23.33 ± 3.02	22.52 ± 3.38	0.26
Systolic BP (mmHg), mean ± SD	111.42 ± 13.08	114.65 ± 13.91	108.2 ± 11.5	0.03
Diastolic BP (mmHg), mean ± SD	70.9 ± 9.15	72.45 ± 8.91	69.35 ± 9.24	0.13
Waist circumference (cm), mean ± SD	74.66 ± 10.13	74.4 ± 11.24	74.92 ± 9.02	0.82
Hip circumference (cm), mean ± SD	97.97 ± 9.15	97.57 ± 10.55	98.37 ± 7.62	0.7
Waist-to-hip ratio, mean ± SD	0.75 ± 0.06	0.75 ± 0.05	0.76 ± 0.06	0.41

Statistical significance *p* < 0.05 (Student’s *t*-test, Mann–Whitney U test, or chi-square test, as appropriate). BMI: body mass index; BP: blood pressure.

**Table 2:** Laboratory and biochemical parameters of the PCOS and control groups.

Parameter	Total	PCOS (*n* = 40)	Control (*n* = 40)	*p*-Value
Fasting glucose (mg/dL)	87.59 ± 8.48	86.78 ± 8.67	88.4 ± 8.32	0.4
Insulin (µU/mL)	7.7 ± 6.7	8.07 ± 7.91	7.33 ± 6.34	0.69
HOMA-IR	1.74 ± 1.05	1.88 ± 1.52	1.61 ± 1.45	0.56
DHEAS (µg/dL)	266.05 ± 114.99	289.22 ± 117.71	242.87 ± 108.77	0.07
Total testosterone (ng/mL)	0.46 ± 0.2	0.52 ± 0.19	0.39 ± 0.19	0.005
LH (IU/L)	9.75 ± 7.29	9.48 ± 5.85	10.03 ± 8.55	0.74
FSH (IU/L)	7.09 ± 4.43	6.65 ± 2.2	7.54 ± 5.88	0.38
LDL (mg/dL)	102.03 ± 30.65	101.36 ± 30.12	102.7 ± 31.54	0.84
HDL (mg/dL)	56.72 ± 11.11	58.53 ± 12.82	54.9 ± 8.86	0.14
Platelets (×10^3^/µL)	274.41 ± 72.85	282.47 ± 76.05	266.35 ± 69.53	0.33
GGT (U/L)	14.25 ± 8.73	13.42 ± 9.51	15.07 ± 7.92	0.4
Albumin (g/L)	4.43 ± 0.29	4.45 ± 0.3	4.4 ± 0.28	0.39
AST (U/L)	20.39 ± 7.3	21.74 ± 8.27	19.03 ± 5.99	0.1
ALT (U/L)	18.99 ± 9.38	20.43 ± 15.95	17.54 ± 9.14	0.51
Total cholesterol (mg/dL)	179.05 ± 39.57	176.52 ± 41.41	181.57 ± 13.68	0.57
Triglycerides (mg/dL)	73.39 ± 34.03	90.6 ± 39.37	56.17 ± 13.68	0.001
Amylase (U/L)	68.92 ± 26.73	74.42 ± 34.55	65.42 ± 15.11	0.24
Lipase (U/L)	29.13 ± 71.54	39.13 ± 101.21	19.37 ± 7.82	0.55
NAFLD liver fat score	−2.11 ± 1.39	−2.09 ± 1.18	−2.14 ± 1.59	0.87
LAP score	12.45 ± 11.48	15.37 ± 14.75	9.52 ± 5.62	0.02
HSI score	47.11 ± 0.53	47.12 ± 0.74	42.9 ± 0.68	0.005
FIB-4	0.58 ± 0.31	0.62 ± 0.38	0.54 ± 0.2	0.27
APRI	0.22 ± 0.1	0.22 ± 0.12	0.2 ± 0.08	0.33
Pancreatic elastography (kPa)	8.19 ± 1.48	8.47 ± 1.63	7.91 ± 1.27	0.09
Hepatic elastography (kPa)	7.71 ± 1.86	7.79 ± 2.05	7.62 ± 1.67	0.69

Statistical significance *p* < 0.05 (Student’s *t*-test or Mann–Whitney U test). HOMA-IR: homeostatic model assessment of insulin resistance; DHEAS: dehydroepiandrosterone sulfate; LAP: lipid accumulation product; HSI: hepatic steatosis index.

**Table 3:** Frequency of hepatic steatosis, fatty pancreas disorder, and metabolic syndrome in the PCOS and control groups.

Variable	Total (*n* = 80)	PCOS (*n* = 40)	Control (*n* = 40)	*p*-Value	OR (95% CI)
Hepatic steatosis, *n* (%)	21 (26.3)	15 (37.5)	6 (15)	0.04	3.40 (1.16–10.00)
Hepatic steatosis grade (mild/moderate), *n*	19/2	13/2	6/0	0.57	—
Metabolic syndrome (IDF), *n* (%)	4 (5)	3 (7.5)	1 (2.5)	0.61	3.16 (0.31–31.78)
Fatty pancreas disorder, *n* (%)	26 (32.5)	19 (47.5)	7 (17.5)	0.008	4.27 (1.53–11.89)
Fatty pancreas disorder grade (mild/moderate), *n*	23/3	16/3	7/0	0.54	—

Statistical significance *p* < 0.05 (chi-square test). IDF: International Diabetes Federation; OR: crude (unadjusted) odds ratio, 95% CI: confidence interval (OR was not calculated for severity subgroups owing to limited cell counts).

**Table 4:** Factors associated with hepatic steatosis independent of PCOS status (*n* = 80).

Characteristic	Hepatic Steatosis Present (*n* = 21)	Hepatic Steatosis Absent (*n* = 59)	*p*-Value
Age (years)	32 ± 8.5	27.5 ± 6.8	0.04
BMI ≥25 kg/m^2^, *n* (%)	15 (71.4)	8 (13.6)	<0.001
Insulin resistance, *n* (%)	3 (14.3)	6 (10.2)	0.69
Metabolic syndrome (IDF), *n* (%)	3 (14.3)	1 (1.7)	0.05
Fatty pancreas disorder, *n* (%)	18 (85.7)	8 (13.6)	<0.001
BMI (kg/m^2^)	26.16 ± 2.17	21.77 ± 2.71	<0.001
Systolic BP (mmHg)	116.66 ± 16.01	109.55 ± 11.45	0.07
Diastolic BP (mmHg)	72.66 ± 9.65	70.27 ± 8.97	0.31
Waist circumference (cm)	85 ± 9.64	70.98 ± 7.43	<0.001
Hip circumference (cm)	105.33 ± 7.46	95.35 ± 8.27	<0.001
Waist-to-hip ratio	0.8 ± 0.05	0.73 ± 0.06	<0.001
Triglycerides (mg/dL)	85.61 ± 48.05	69.03 ± 26.59	0.05
LAP score	22.73 ± 15.95	8.79 ± 6.31	0.001
HSI score	38.39 ± 3.14	34.6 ± 4.79	0.001
FIB-4	0.65 ± 0.37	0.55 ± 0.27	0.17
APRI	0.22 ± 0.12	0.21 ± 0.09	0.49
Hepatic elastography (kPa)	8.49 ± 2.21	7.42 ± 1.66	0.02
Pancreatic elastography (kPa)	8.95 ± 1.76	7.92 ± 1.27	0.005

Statistical significance *p* < 0.05. Variables that did not reach significance (fasting glucose, insulin, HOMA-IR, DHEAS, total testosterone, LH, FSH, LDL, HDL, platelets, GGT, albumin, AST, ALT, total cholesterol, amylase, lipase, NAFLD score) are summarized in the text.

**Table 5:** Factors associated with fatty pancreas disorder independent of PCOS status (*n* = 80).

Characteristic	Fatty Pancreas Disorder Present (*n* = 26)	Fatty Pancreas Disorder Absent (*n* = 54)	*p*-Value
Age (years)	31.1 ± 8.1	27.6 ± 7.1	0.04
BMI ≥25 kg/m^2^, *n* (%)	17 (65.4)	6 (11.1)	<0.001
Insulin resistance, *n* (%)	5 (19.2)	4 (7.4)	0.42
Hepatic steatosis, *n* (%)	18 (69.2)	3 (5.6)	<0.001
Metabolic syndrome (IDF), *n* (%)	4 (15.4)	0 (0)	0.009
BMI (kg/m^2^)	25.71 ± 2.26	21.58 ± 2.71	<0.001
Systolic BP (mmHg)	117.08 ± 15.13	108.71 ± 19.12	0.02
Diastolic BP (mmHg)	73.11 ± 9.7	69.83 ± 8.77	0.13
Waist circumference (cm)	81.46 ± 10.63	71.38 ± 8.13	<0.001
Hip circumference (cm)	103.04 ± 7.62	95.53 ± 8.88	<0.001
Waist-to-hip ratio	0.78 ± 0.07	0.74 ± 0.05	0.001
Triglycerides (mg/dL)	91.15 ± 46.55	64.83 ± 21.77	0.01
Hepatic elastography (kPa)	8.95 ± 1.77	7.83 ± 1.17	0.006
Pancreatic elastography (kPa)	8.31 ± 2.17	7.41 ± 1.64	0.04

Statistical significance *p* < 0.05. Variables that did not reach significance (fasting glucose, insulin, HOMA-IR, DHEAS, total testosterone, LH, FSH, LDL, HDL, platelets, GGT, albumin, AST, ALT, total cholesterol, amylase, lipase) are summarized in the text.

**Table 6:** Correlation of hepatic and pancreatic elastography values with age and BMI (*n* = 80).

Variable	Statistic	Hepatic Elastography (kPa)	Pancreatic Elastography (kPa)
Age	r/*p*	−0.12/0.29	0.07/0.53
BMI	r/*p*	0.40/<0.001	0.35/0.003

Pearson correlation coefficient; statistical significance *p* < 0.05.

**Table 7:** Multivariable logistic regression analysis of factors associated with fatty pancreas disorder (*n* = 80).

Variable	*p*-Value	Exp(B)	95% CI Lower	95% CI Upper
BMI	0.000	1.765	1.323	2.354
Age	0.226	1.069	0.960	1.190
PCOS status	0.048	5.474	1.018	29.426
Systolic BP	0.150	1.056	0.980	1.138
Triglycerides	0.259	1.015	0.989	1.042

Statistical significance *p* < 0.05. CI: confidence interval; BP: blood pressure. Dependent variable: fatty pancreas disorder (present = 1/absent = 0).

## Data Availability

The data presented in this study are available from the corresponding author upon reasonable request.
